# Expression level of a *flavonoid 3′-hydroxylase* gene determines pathogen-induced color variation in sorghum

**DOI:** 10.1186/1756-0500-7-761

**Published:** 2014-10-27

**Authors:** Hiroshi Mizuno, Takayuki Yazawa, Shigemitsu Kasuga, Yuji Sawada, Jun Ogata, Tsuyu Ando, Hiroyuki Kanamori, Jun-ichi Yonemaru, Jianzhong Wu, Masami Yokota Hirai, Takashi Matsumoto, Hiroyuki Kawahigashi

**Affiliations:** National Institute of Agrobiological Sciences, Agrogenomics Research Center, 1-2, Kannondai 2-chome, Tsukuba, Ibaraki, 305-8602 Japan; Hitachi Government & Public Corporation System Engineering Ltd, Koto-ku, Tokyo, Japan; Faculty of Agriculture, Shinshu University, 8304 Minami-minowa, Nagano, 399-4598 Japan; RIKEN Center for Sustainable Resource Science, Yokohama, Kanagawa, 230-0045 Japan

**Keywords:** Apigeninidin, 3-deoxyanthocyanidin, Luteolinidin, Transcriptome

## Abstract

**Background:**

Sorghum (*Sorghum bicolor* L. Moench) accumulates 3-deoxyanthocyanidins and exhibits orange to purple coloration on parts of the leaf in response to infection with the fungus *Bipolaris sorghicola*. We aimed to identify the key genes determining this color variation.

**Results:**

Sorghum populations derived from Nakei-MS3B and M36001 accumulated apigeninidin, or both apigeninidin and luteolinidin, in different proportions in lesions caused by *B. sorghicola* infection, suggesting that the relative proportions of the two 3-deoxyanthocyanidins determine color variation. QTL analysis and genomic sequencing indicated that two closely linked loci on chromosome 4, containing the *flavonoid 3′-hydroxylase* (*F3′H*) and *Tannin1* (*Tan1*) genes, were responsible for the lesion color variation. The *F3′H* locus in Nakei-MS3B had a genomic deletion resulting in the fusion of two tandemly arrayed *F3′H* genes. The recessive allele at the *Tan1* locus derived from M36001 had a genomic insertion and encoded a non-functional WD40 repeat transcription factor. Whole-mRNA sequencing revealed that expression of the fused *F3′H* gene was conspicuously induced in purple sorghum lines. The levels of expression of *F3′H* matched the relative proportions of apigeninidin and luteolinidin.

**Conclusions:**

Expression of *F3′H* is responsible for the synthesis of luteolinidin; the expression level of this gene is therefore critical in determining color variation in sorghum leaves infected with *B. sorghicola*.

**Electronic supplementary material:**

The online version of this article (doi:10.1186/1756-0500-7-761) contains supplementary material, which is available to authorized users.

## Background

Sorghum (*Sorghum bicolor* L. Moench) is a rich source of phytochemicals, including certain 3-deoxyanthocyanidins [1], dhurrin [2], and sorgoleone [3]. 3-deoxyanthocyanidins are not commonly found in higher plants [4], but sorghum accumulates them in response to pathogen infection [1, 5–7]. One 3-deoxyanthocyanidin, luteolinidin, is toxic to fungi and accumulates at increased levels in sorghum lines resistant to the anthracnose fungus [5, 8]. Sorghum that accumulates 3-deoxyanthocyanidins exhibits various changes in coloration after infection with *B. sorghicola* [6]. The sorghum *REDforGREEN* mutant accumulates a >1000-fold higher amounts of the 3-deoxyanthocyanidins luteolinidin and apigeninidin (and variants) than the wild type and exhibits intense red–purple color of the leaves [6, 9]. However, the enzymes required for 3-deoxyanthocyanidin synthesis have not been fully identified, and the key genes required for detemining color variation remain to be elucidated.

Functional genomic studies of sorghum began after its genome sequencing was completed in 2009 [10, 11]. Whole-genome sequencing of sorghum BTx623 has revealed that many genes are duplicated and tandemly arrayed [10]. Each gene may have developed different functions related to a particular biochemical reaction. The sequence similarity of these duplicated genes makes it difficult to distinguish the expression of gene members of this family by using polymerase chain reaction (PCR)- or oligonucleotide array-based technology. Given the rapid progress of next-generation sequencing technology, shotgun sequencing of whole transcripts—so called RNA-seq—has been used for the profiling of gene expression in sorghum in response to infection with the fungus *Bipolaris sorghicola*, the cause of target leaf spot [12, 13]. 3-deoxyanthocyanidin biosynthesis after infection with *B. sorghicola* occurs through the coordinated expression of genes encoding the catalysts of sequential reactions; these catalysts include phenylalanine ammonia lyase, trans-cinnamate 4-monooxygenase, 4-coumarate:CoA ligase, chalcone synthase (CHS), chalcone isomerase (CHI), dihydroflavonol 4-reductase (DFR), and putative anthocyanidin reductase [12]. *De novo* transcriptome assembly has revealed that transcripts derived from *B. sorghicola* induce a defense response in sorghum [13]. Transcriptome analysis is a powerful tool for identifying the key genes expressed among family members.

Here, we aimed to identify the key genes detemining color variation in sorghum. For this purpose, we used sorghum populations derived from Nakei-MS3B (which has purple *B. sorghicola* lesions) × M36001 (which shows no color change with *B. sorghicola* infection); this population shows a gradation of different colors. We performed a metabolic analysis to identify accumulated pigments, a quantitative trait locus (QTL) analysis to map candidate genes, and whole mRNA sequencing to comprehensively identify the genes expressed. We found that the expression levels of a particular *flavonoid 3′-hydroxylase* (*F3′H*) gene on chromosome 4 matched the relative proportions of the 3-deoxyanthocyanidins apigeninidin and luteolinidin, and this gene was thus responsible for the gradual variation of colors in sorghum leaves infected with *B. sorghicola*.

## Methods

### Plant materials and phenotyping

The sorghum cultivar Nakei-MS3B, and the M36001 were used as parents. A mapping population was established from a cross between these cultivars. For the plant color test, at Shinshu University in Nagano, Japan, in 2011, the F2 population was grown and inoculated with barley seeds colonized by *Bipolaris sorghicola.* At Tsukuba, Ibaraki, Japan, in 2012, the F3 populations were subjected to high-density genetic mapping and mRNA-seq analysis. Accumulated pigments were quantified by using LC-MS/MS as described previously [14].

### Marker development and genetic mapping

A mapping population was established from a cross between the sorghum cultivars Nakei-MS3B and M36001. We used 150 F2 progeny; the 122 progeny with color changes in their lesions were used for bulk mapping of purple or orange leaf color, with an analysis of 172 sorghum SSR markers as described previously [15]. The major SSR markers used for the genetic mapping of plant color are shown in Additional file 1: Table S1. QTL analysis was performed for the entire population by using Windows QTL Cartographer ver. 2.5 (http://statgen.ncsu.edu/qtlcart/WQTLCart.htm). The F2 intercross algorithm and default linkage criteria [LOD (logarithm (base 10) of odds) 3.0 and 50 cM maximum distance) were applied. The Kosambi function was used to establish genetic distances.

### Construction and screening of a sorghum BAC library

BAC (bacterial artificial chromosome) libraries were constructed from young leaves of Nakei-MS3B; they contained 39,267 (average insert size 134 kb) clones, respectively. We used conventional methods, namely a partial DNA digest with *Hin*dIII enzyme, size fractionation of high-molecular-weight DNA by pulsed-field gel electrophoresis (CHEF; Bio-Rad Laboratories, USA), and vector ligation (pIndigoBAC-5; Epicentre Biotechnologies Madison, WI, USA) and transformation into *E. coli* (DH10B strain). Positive BAC clones covering the region of the *F3′H* gene were screened from each library by using tightly linked DNA markers through PCR amplification, and subjected to shotgun sequencing to give approximately 10-fold sequence coverage using a previously described method [16]. A BAC clone containing inserts from the *F3′H* region—namely MS3B_108E24 (183 kb)—from Nakei-MS3B was found by PCR analysis by using SB20978 and SB20980 (Additional file 1: Table S1). The BAC sequences were produced by Sanger shotgun sequencing of subclones followed by assembly of the shotgun sequences. The sequences of candidate genes were obtained from the sorghum genome database (http://www.plantgdb.org) and used for gene expression analysis.

### RNA-seq

To extract RNA from each plant tissue, five biological replicates were collected, immediately frozen in liquid nitrogen, and mixed to minimize the effect of transcriptome unevenness among plants. Total RNA was extracted by using an RNeasy Plant kit (Qiagen, Hilden, Germany). RNA quality was calculated with a Bioanalyzer 2100 algorithm (Agilent Technologies, Palo Alto, CA, USA); high-quality (RNA Integrity Number >8) RNA was used. Total RNA samples (10 μg) were subjected to cDNA construction for Illumina sequencing, in accordance with the protocol for the mRNA-Seq sample preparation kit (Illumina, San Diego, CA, USA). Oligo (dT) magnetic beads were used to isolate poly (A) RNA from the total RNA samples. The mRNA was fragmented by heating at 94°C for 5 min. First-strand cDNA was synthesized by using random hexamer primers at 25°C for 10 min, 42°C for 50 min, and 70°C for 15 min. After the first strand had been synthesized, dNTPs, RNaseH, and DNA polymerase I were added to synthesize second-strand DNA for 2.5 h at 16°C. The ends of double-stranded cDNA were repaired by using T4 DNA polymerase and Klenow DNA polymerase and phosphorylated by using T4 polynucleotide kinase. A single “A” base was added to the cDNA molecules by using Klenow exonuclease, and the fragments were ligated to the paired end (PE) adapters from the Illumina mRNA-Seq kit. cDNA with 200 ± 25-bp fragments was collected. The purified cDNA was amplified by using 15 cycles of PCR at 98°C for 10 s, 65°C for 30 s, and 72°C for 30 s using PE1.0 and PE2.0 primers.

### Bioinformatics

We used an in-house program to trim out low-quality nucleotides (<Q15) from both the 5′- and the 3′-ends of the reads until a stretch of 3 bp or more of high-quality (≥Q15) nucleotides appeared. Adaptors were also trimmed out by using Cutadapt version 1.0 (http://code.google.com/p/cutadapt/). We used Bowtie 2 version 2.0.0 beta6 [17] to align the reads against sorghum rRNA gene sequences downloaded from the Plant Repeat Database [18]; aligned reads were removed. The reads were deposited in the DDBJ (DNA Data Bank of Japan) Sequence Read Archive (Accession No. DRA001265).

The reads were aligned to the sorghum reference genome of BTx623 [10] by using Bowtie 2, SAMtools version 0.1.18 [19], and TopHat version 2.0.4 [20]. RPKM (Reads Per Kilobase of exon model per Million mapped reads) [21] values were calculated for each transcript annotated in Phytozome [22] or assembled by using Cufflinks version 2.0.0 [23]. Transcripts that were differentially expressed between cutting-stress samples and control samples were detected by using a G-test with a false discovery rate threshold of 0.1%.

The Sb04g024710 gene sequence was aligned to genes from Phytozome and Cufflinks by using BLAST + version 2.2.26 [24], and the top 50 hits were considered to be Sb04g024710 paralogs. A heatmap of Sb04g024710 paralogs was generated by using R (http://www.R-project.org/) package gplots version 2.10.1 (http://cran.r-project.org/web/packages/gplots/index.html) with log2 values of the RPKM fold changes. Reads mapped to genes in the *F3′H* region were visualized by using Integrative Genomics Viewer [25].

## Results

### Identification of pigments in sorghum exhibiting different-colored lesions

**Figure 1:**
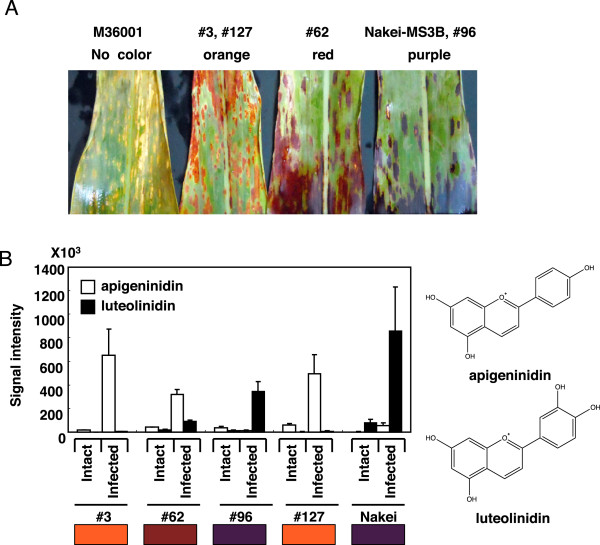
**Coloration of sorghum leaves after infection with** ***Bipolaris sorghicola*** **. (A)** Nakei-MS3B (right) has purple lesions and M36001 (left) has no change in color. Populations derived from Nakei-MS3B × M36001 had purple (#96), orange (#3, #127) or red (#62) lesions. **(B)** Quantification of accumulated pigments by LC-MS/MS. The “orange” lines (#3, #127) accumulated apigeninidin, whereas the “purple” lines (#96, Nakei-MS3B) accumulated luteolinidin. The “red” line (#62) accumulated both pigments. Box indicates color exhibited by lesions in each sorghum line.

Sorghum leaves exhibit various colors upon infection with *Bipolaris sorghicola*. Sorghum populations derived from a cross between Nakei-MS3B and M36001 had spots of purple (Nakei-MS3B, #96), red (#62), or orange (#3, #127), or no color change (M36001) (Figure 1A). Sorghum plants produce the 3-deoxyanthocyanidins apigeninidin and luteolinidin in these lesions [12]. Accumulation of these pigments was confirmed by using thin layer chromatography and high performance liquid chromatography (data not shown). In each line, the color pigments in the lesions were further analyzed by using liquid chromatography – mass spectrometry/mass spectrometry (LC-MS/MS) (Figure 1B), which was confirmed by the retention time and MS and MS/MS of authentic compounds [14]. Apigeninidin and luteolinidin were barely detected before infection but were clearly produced after infection. The purple lesions on Nakei-MS3B and line #96 contained luteolinidin and a small amount of apigeninidin, whereas the orange lesions on line #3 contained only apigeninidin. The red lesions on line #62 contained both luteolinidin and apigeninidin in relatively small proportions. Therefore, the relative proportions of luteolinidin and apigeninidin determined lesion color upon infection with *Bipolaris sorghicola.*

### Mapping of QTLs responsible for color variation in *B. sorghicola*lesions on sorghum

**Figure 2:**
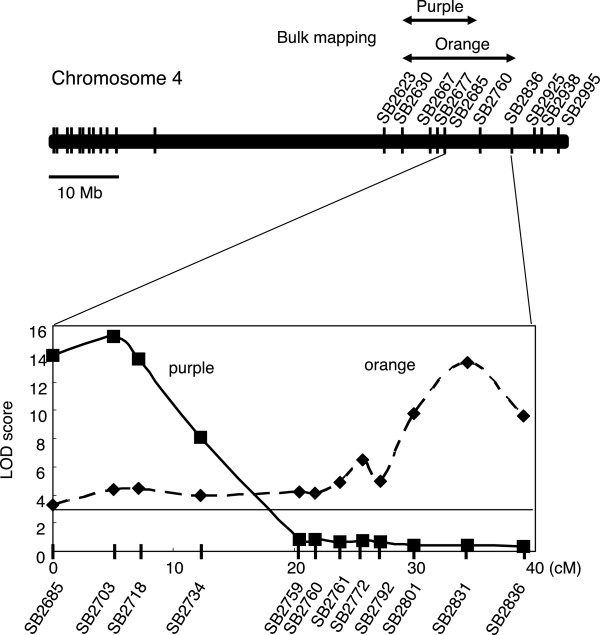
**Quantitative trait locus (QTL) analysis for color variation.** (Upper) The purple or orange leaf color locus was mapped on chromosome 4 as a single locus. (Lower) QTL likelihood curves of logarithm of odds (LOD) scores for color variation of lesions on sorghum leaves after infection with *Bipolaris sorghicola* (purple, solid line; orange, dashed line) show significant regions on chromosome 4. Using the F2 population, the predicted regions were segregated into a region for purple and one for orange. Genetic distances (in centimorgans) between markers are indicated on the *X* axis. Vertical line corresponds to the critical LOD value.

We identified QTLs determining color variation in the sorghum lesions by using a population derived from Nakei-MS3B × M36001. The F_2_ population segregated for 150 individuals with and without color pigmentation change at a frequency of 122:28 (*χ*^2^ = 3.20; *P* = 0.073 for a 3:1 segregation ratio, chi-squared test). Because our aim was to elucidate the genes responsible for color variation, we subjected the 122 colored-lesion F_2_ progeny to further analysis. The ratio of the pigments in the lesions was purple to red to orange = 24:67:31 (*χ*^2^ = 1.262; *P* = 0.532 for 1:2:1 segregation ratio, chi-squared test), suggesting that color variation was controlled by a single semi-dominant locus. Bulk mapping revealed a clear bias toward purple or orange lesion color between simple sequence repeat (SSR) markers SB2623 (44.80 Mb) and SB2925 (66.54 Mb) on chromosome 4, indicating that color variation–related genes were present at a single locus (Figure 2, upper panel). We then subjected 150 F_2_ plants to genetic mapping, which revealed that the predicted regions were segregated into two regions, between SB2685 (53.07 Mb) and SB2734 (56.42 Mb) for purple and between SB2760 (57.96 Mb) and SB2836 (62.14 M b) for orange (Figure 2). Further mapping showed that the candidate genes responsible for purple were located in an 880-kb region between SB2703 (54.15 Mb) and SB2710 (55.03 Mb); those for orange were located in a 2.09-Mb region between SB2792 (60.05 Mb) and SB2836 (62.14 Mb).

### *F3′H*locus

At the first locus, which was responsible for the purple and was located between the SSR markers SB2703 and SB2710, 85 genes were annotated in Phytozome [22]. At this locus, three *F3′H* genes (Sb04g024710, Sb04g024730, Sb04g024750) are tandemly arrayed on the BTx623 genome in the Phytozome annotation. *F3′H*s are enzymes that introduce a hydroxyl group at the 3′ position of ring B of the flavonoid. We did not perform a complementation experiment on the *F3′H* proteins encoded by these genes in sorghum, but the proteins encoded by Sb04g024710 and Sb04g024750 (previously named *SbF3′H2* and *SbF3′H1*, respectively) have *F3′H* activity in the *tt7* mutant of Arabidopsis to produce 3′-hydroxylated flavonoids [26]. As the other genes located at the locus were not likely to be involved in flavonoid synthesis, we focused only on these *F3′H* genes.

**Figure 3:**
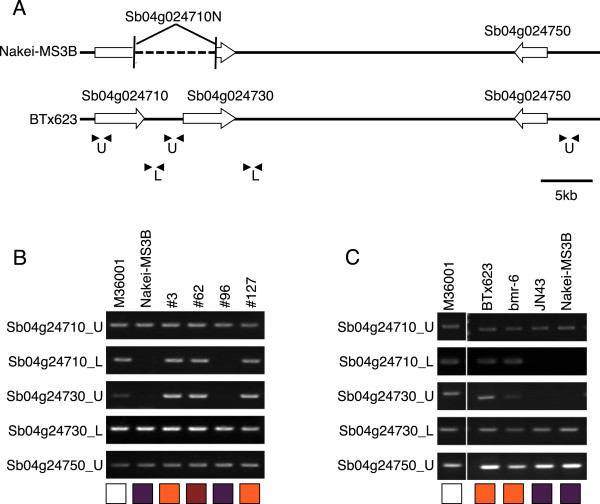
**Genomic deletion at the** ***F3′H*** **locus. (A)** Genomic structure of *F3′H* loci. Three *F3′H* genes are tandemly duplicated in BTx623. Nakei-MS3B has a genomic deletion affecting Sb04g024710 and Sb04g024730, resulting in fusion of the open reading frames of these *F3′H* genes (Sb04g024710N). **(B, C)** Genomic PCR analysis. Genomic PCR of F2 lines derived from Nakei-MS3B × M36001 **(B)** and five sorghum varieties **(C)**. Boxes at bottom indicate colors on areas of each sorghum plant infected with *Bipolaris sorghicola*. Positions of the PCR primers are indicated by arrowheads in panel **A**.

The genomic sequences of the *F3′H* loci were compared in BTx623 (orange) and Nakei-MS3B (purple). BTx623 had three tandemly arrayed *F3′H* genes (Sb04g024710/*SbF3′H2*, Sb04g024730/*SbF3′H3*, and Sb04g024750/*SbF3′H1*), whereas Nakei-MS3B had a genomic deletion flanked by the 5*′*-region of Sb04g024710/*SbF3′H2* and the 3*′*-region of Sb04g024730/*SbF3′H3*, resulting in only two *F3′H* genes (an Sb04g024710–30 fused gene named Sb04g024710N and Sb04g024750/*SbF3′H1*) at the locus (Figure 3A). The fused *F3′H* protein in Nakei-MS3B had two amino acid substitutions in the C-terminal region, namely K503M and A507T (Additional file 2: Figure S1). The deletion was detected in lines exhibiting purple lesions (Nakei-MS3B, #96), but not in lines exhibiting orange ones or no color change (#3, #127, M36001; Figure 3B), suggesting that the deletion was inherited from Nakei-MS3B. A heterozygous line (#62) with red lesions contained both alleles (Figure 3B). Genomic PCR analysis confirmed that accessions exhibiting purple lesions after *Bipolaris sorghicola* infection (i.e. JN43 and Nakei-MS3B) had the deletion, but those exhibiting no color change or orange lesions (i.e. BTx623, bmr-6, and M36001; Figure 3C) did not. This result suggested that there was an association between genomic deletion at the *F3′H* locus and lesion color.

We compared the genomic sequences of the region upstream of Sb04g024710 between BTx623 and Nakei-MS3B. Two nucleotide substitutions (at positions -144 and -664), and one insertion/deletion (at position -661) were found in the 1000-bp region upstream of the transcription start site of the Sb04g024710 gene (Additional file 3: Figure S2). We searched for candidate *cis*-regulatory elements by using the PLACE (Plant *cis*-regulatory DNA Elements) [27] program. CTCTT (found only in Nakei-MS3B at position -664) is one of the consensus sequence motifs in promoters activated in the infected cells of root nodules in *Vicia faba*, *Glycine max* [28–30]. CACT (found only in BTx623 at position -144) is a key component of Mem1 (mesophyll expression module 1), which is found in the *cis*-regulatory element of phosphoenolpyruvate carboxylase (ppcA1) in the C4 dicot *Flaveria trinervia* [31]. GCCAC (found only in Nakei-MS3B at position -644 antisense) is a promoter motif involved in light-induced gene expression in Arabidopsis and rice [32, 33].

### *Tannin1*locus

At the second locus, which was responsible for the orange and was located between the SSR markers SB2792 and SB2836, we found 243 genes annotated in Phytozome. Among these, the *Tannin1* (*Tan1*) gene encoding a WD40 repeat transcription factor (Sb04g031730). Tan1 controls tannin biosynthesis in sorghum, and transforming the sorghum *Tan1* open reading frame into a nontannin Arabidopsis mutant restores the tannin phenotype [34]. *Tan1* derived from M36001 (which has no color change upon fungus infection) had a 10-bp insertion (CGGGCAGCGG) in the exon region that caused a frame shift at position 921 nt (307aa) (Additional file 4: Figure S3), suggesting that this allele encoded a non-functional transcription factor. M36001, #127, and #3 did not accumulate tannin in the seeds (Table 1). *Tan1* is similar to *PAC1* (*Pale aleurone color1*), which encodes a regulator of the maize anthocyanin pathway [35].

**Table 1:** **Associations of phenotypes with** ***F3′H*** **and** ***Tan1*** **genotypes**

	Genotype		Phenotype	
	***F3′H***	***Tan1***	Color of leaf lesion	Accumulation of tannin in seeds*
Nakei-MS3B	*F3′H*/*F3′H*	*Tan1/Tan1*	Purple	yes
#96	*F3′H/F3′H*	*Tan1/tan1-b*	Purple	yes
#62	*F3′H/f3′h*	*Tan1/tan1-b*	Red	yes
#3	*f3′h/f3′h*	*tan1-b/tan1-b*	Orange	no
#127	*f3′h/f3′h*	*tan1-b/tan1-b*	Orange	no
M36001	*f3′h/f3′h*	*tan1-b/tan1-b*	No color change	no

*Accumulation of tannin was determined by the bleach test.

*F3′H*: *flavonoid 3′-hydroxylase; Tan1*: *Tannin1.*

As other candidate genes at the locus, we located genes encoding putative MYB transcription factors (Sb04g031030 and Sb04g031820) or MYB-related proteins (Sb04g030510 and Sb04g031110). The putative MYB transcription factor gene (Sb04g031820) was highly expressed, but its expression level did not change after wounding stress (Additional file 5: Table S2). Another putative MYB transcription factor gene (Sb04g031030) and MYB-related protein genes (Sb04g030510 and Sb04g031110) were barely expressed (Additional file 5: Table S2). Therefore, we focused on the *Tan1* (Sb04g031730) gene.

### Transcriptome analysis of sorghum exhibiting different-colored lesions

**Figure 4:**
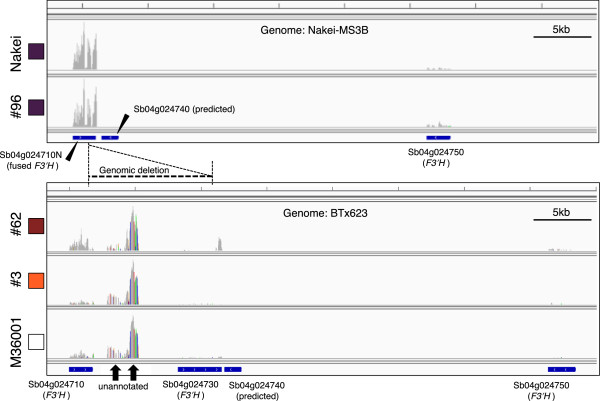
**Results of mapping of short reads by using mRNA-seq at the** ***F3′H*** **locus.** Reads from Nakei-MS3B (Nakei) and #96 were mapped on the Nakei-MS3B genome, which has two *flavanone 3′-hydroxylase* (*F3′H*) genes (Sb04g024710N and Sb04g024750) because of the genomic deletion. Reads from lines #62, #3, and M36001 were mapped on the BTx623 genome, which has three *F3′H* genes (Sb04g024710, Sb04g024730, and Sb04g024750). Line #62 has heterozygous alleles, but only the allele derived from M36001 is shown. *F3′H* expression was strongly induced in sorghum lines with purple lesions (Sb04g024710N: Nakei and #96) and intermediately in the line with red lesions (Sb04g024710: #62), but it was barely expressed in those with orange lesions (Sb04g024710: #3) or no color change (Sb04g024710: M36001). Arrows indicate transcription from the unannotated region between Sb04g024710 and Sb04g024730.

To determine which *F3′H* was responsible for the pigmentation, we used RNA-seq to identify genes that were differentially expressed after cutting stress of sorghum leaves. *F3′H* (Sb04g024710N) was strongly induced in sorghum lines with purple lesions and intermediately in those with red lesions, but it was barely expressed in those with orange lesions (Figure 4). The high level of expression was consistent with the accumulation of luteolinidin (Figure 1B).

**Figure 5:**
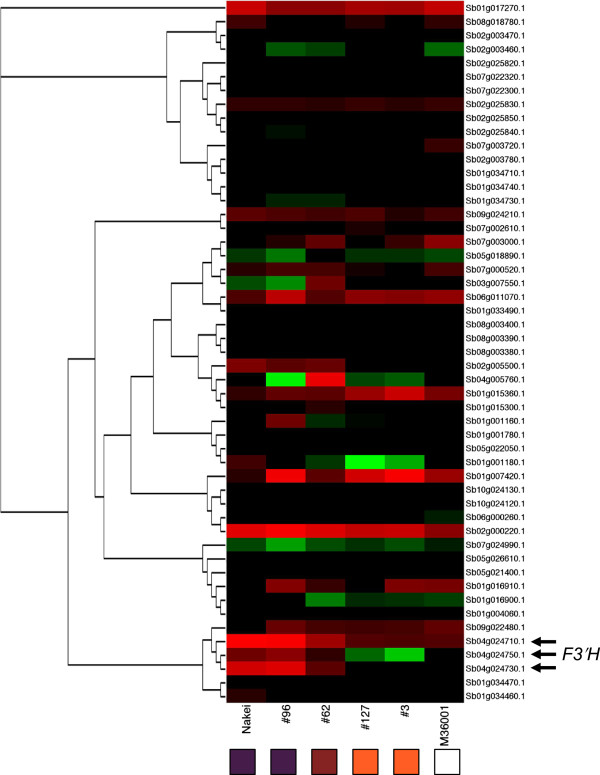
**Differential expression of** ***F3′H*** **genes.** Expression of the top 50 genes with high levels of identity to the *F3′H* gene Sb04g024710 was used to create this heatmap (red, upregulation; green, downregulation). *F3′H* (arrows) expression was induced in Nakei-MS3B and lines #96 and #62. Boxes at the bottom indicate colors of areas of each sorghum plant infected with *Bipolaris sorghicola*.

We then used RNA-seq to compare the relative expression levels of *F3′H* genes among 50 family genes with high levels of identity to Sb04g024710. Expression of *F3′H* (Sb04g024710N) was exclusively induced in sorghum with purple lesions (Figure 5). We therefore considered that expression of an *F3′H* gene (Sb04g024710N) is responsible for the synthesis of luteolinidin and thus plays a critical role in color variation in *B sorghicola* spots on sorghum leaves.

## Discussion

We aimed to elucidate the key genes determining color variation in sorghum infected with *B. sorghicola*. We used sorghum populations derived from Nakei-MS3B (purple lesions) × M36001 (no color change in lesions), which showed graduated changes in lesion color. Metabolic analysis suggested that the relative proportions of the apigeninidin and luteolinidin determined color variation (Figure 1). QTL analysis (Figure 2) and genomic sequencing (Figure 3, Additional file 3: Figure S2, and Additional file 4: Figure S3) suggested that two loci, containing the *F3′H* gene and the *Tan1* transcription factor gene, were responsible for the color variation (Table 1). Finally, mRNA-seq suggested that the expression of one *F3′H* gene (Sb04g024710N) was particularly induced in sorghum lines with purple lesions (Figures 4 and 5). We therefore concluded that *F3′H* is responsible for synthesis of luteolinidin, and that its expression level is a critical determinant of color variation in sorghum.

### *F3′H*in the 3-deoxyanthocyanidin pathway

**Figure 6:**
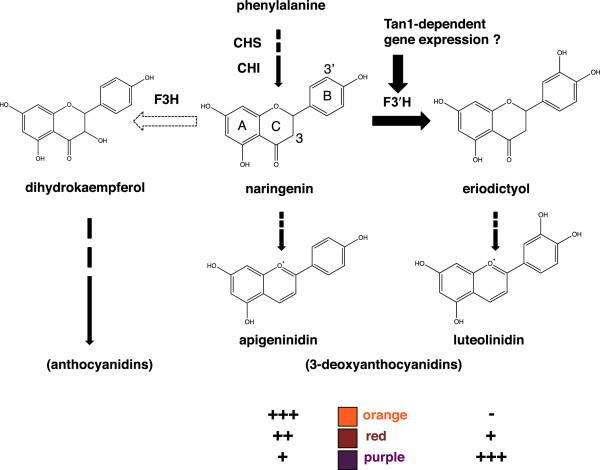
**Role of** ***F3′H*** **in the 3-deoxyanthocyanidin pathway.** Flavonoid compounds share the same basic skeleton of a flavan nucleus consisting of two aromatic rings with six carbon atoms (rings A and B), which are interconnected by a heterocyclic ring with three carbon atoms (ring C). Flavanone 3′-hydroxylase (*F3′H*) introduces a hydroxyl group at the 3′ position of ring B of naringenin. Expression of *F3′H* thus adds the step that leads to the production of luteolinidin. The expression level of *F3′H* matches the color exhibited on the areas of each sorghum plant infected with *Bipolaris sorghicola*. High-level expression of *F3′H* may depend on the action of Tan1 transcription factor. Flavanone 3-hydroxylase (F3H) catalyzes C-3 hydroxylation of the C ring of naringenin, leading ultimately to anthocyanidin production. However, *F3H* was not expressed in the sorghum cultivars we studied (dotted arrow). Naringenin is the common intermediate for apigeninidin, luteolinidin, or the anthocyanidins.

The difference between the chemical formulae of apigeninidin (4′-hydroxylated) and luteolinidin (3′,4′-hydroxylated) is the hydroxylation at the 3′ position of ring B in luteolinidin (Figures 1 and 6). Our QTL analysis (Figure 2) and RNA-seq (Figures 4 and 5) analysis suggested that *F3′H* was responsible for the color variation. *F3′H* enzyme hydroxylates the 3′ position of the B-ring of naringenin to produce eriodictyol [36–38]. Sorghum *F3′H* (Sb04g024710, previously named *SbF3′H2*)-encoded proteins have *F3′H* activity *in vivo* to produce 3′-hydroxylated flavonoids [8, 26]. Therefore, we consider that expression of *F3′H* added this step of hydroxylation at the 3′-position of ring B of naringenin; consequently, an additional step led to the production of luteolinidin in the 3-deoxyanthocyanidin pathway (Figure 6).

The 3-deoxyanthocyanidins luteolinidin and apigeninidin are unique flavonoids that are not commonly found in higher plants [4]. Sorghum accumulates 3-deoxyanthocyanidins synthesized from phenylalanine through naringenin as a common intermediate of anthocyanidins (Figure 6). Anthocyanidins are synthesized by the action of flavanone 3-hydroxylase (F3H), which in maize hydroxylates the 3 position of ring C of naringenin [36] (Figure 6). Sorghum *F3H1* (Sb06g031790.1) was not expressed in Nakei-MS3B or M36001 (Additional file 5: Table S2), as was found in a previous study of BTx623 [12, 39]. This lack of F3H activity is the critical determinant of the pathway to production of the unique 3-deoxyanthocyanidin flavonoids, instead of anthocyanidins, in sorghum (Figure 6). We therefore consider that naringenin is the branching point of the metabolic pathway to apigeninidin, luteolinidin, or the anthocyanidins.

### What determines the activity of *F3′H*in sorghum tissues?

#### (1) *F3′H*locus

*F3′H* (Sb04g024710N) was highly expressed in Nakei-MS3B exhibiting purple lesions (Figures 4 and 5). *F3′H* (Sb04g024710) in M36001 and *F3′H* (Sb04g024750) in both lines were also expressed, but the expression levels were not as high as that of Sb04g024710N in Nakei-MS3B (Figure 4). This suggests that the expression level of *F3′H*s determines the activity of F3′H in the lesions. We considered that high-level expression was related mainly to the genomic deletion (Figure 3A), as the deletion was commonly inherited from Nakei-MS3B (Figure 3B) and was found in sorghum cultivars with purple lesions (Figure 3C). In the deleted region between Sb04g024710 and Sb04g024730 in lines #3, #62, and M36001, RNA-seq analysis revealed transcription from two unannotated regions (Figure 3B). The upstream transcript had 90% identity with that encoding the DNA-binding protein of *Zea mays* LOC100281685, and the downstream transcript encoded a heparin-α-glucosamide N-acetyltransferase-like protein similar to that of *Setaria italica* (LOC101768299). This transcription might inhibit proximal *F3′H* expression in lines #3, #62 (which had heterozygous alleles), and M36001, and the inhibition might be released by the genomic deletion in Nakei-MS3B and lines #96 and #62. Nucleotide substitutions or insertion/deletion, or both, in the region upstream of *F3′H* (Additional file 3: Figure S2) might affect the binding affinity of transcription factors to the promoter of *F3′H*, thus also changing the expression level of *F3′H*. In addition to these changes in expression level, amino acid substitutions in the C-terminal region of the *F3′H* protein (Figure 3A and Additional file 2: Figure S1) might change the enzymatic activity of *F3′H*. These factors may synergistically affect total *F3′H* activity (Sb04g024710N in Nakei-MS3B or Sb04g024710 in M36001, and Sb04g024750 in both lines) in sorghum tissues and thus determine the relative proportions of apigeninidin and luteolinidin.

#### (2) Tan1 transcription factor

Our QTL analysis suggested that the locus containing *Tan1* was responsible for color variation (Figure 2). Tan1 regulates the expression of genes encoding enzymes in the tannin or anthocyanin pathway, or both pathways, in the sorghum seed coat; these enzymes include CHS, CHI, F3H, DFR, ANS (anthocyanin synthase), and LAR (leucoanthocyanidin reductase) [34]. We hypothesized that Tan1 also controls the expression of *F3′H* (Figure 6). Expression of *F3′H* (Sb04g024750; this gene is common to all the sorghum lines used in this study) was higher in Nakei-MS3B (*Tan1/Tan1)* and #96 (*Tan1*/*tan1-b*) than in #3 and #127 (both of which had the *tan1-b/tan1-b* allele; Table 1, Additional file 5: Table S2); several lines (*f3′h/ f3′h*, *Tan1/Tan1;* data not shown) had reddish lesions, unlike the orange lesions in #3 and #127 (*f3′h/ f3′h*, *tan1-b/tan1-b*). This suggests that Tan1 enhances the expression of *F3′H* in the leaf*. Tan1* (Sb04g031730) was expressed in all sorghum lines used in this study (RPKM: 3.0–10.6; Additional file 5: Table S2), but the 10-bp insertion (CGGGCAGCGG) in the exon region of the *tan1-b* allele caused a frame shift in the encoded protein (Additional file 4: Figure S3). BTx623 [34], M36001, #127, and #3 (all of which had the *tan1-b/tan1-b* allele) did not accumulate tannin in their seeds (Table 1), suggesting that this insertion is a common feature of the alleles encoding non-functional Tan1 transcription factors. As *F3′H* (Sb04g024710) was slightly expressed in M36001, #127, and #3 (Figure 4), Tan1 is not essential for *F3′H* expression in the leaf. We therefore consider that although the *Tan1* allele is not essential for *F3′H* expression, Tan1 enhances *F3′H* expression and thus contributes to the generation of color variation in sorghum leaves.

Tan1 is a WD40-repeat protein. As the expression of anthocyanin biosynthetic genes is regulated through a complex of WD40-repeat proteins, MYB transcription factors (TFs), and basic helix-loop-helix (bHLH) TFs [40], Tan1 may form a complex with MYB TFs and bHLH TFs. Sorghum *F3′H* is regulated by a MYB protein encoded by *yellow seed1* (*y1*) in the seed coat [41], an ortholog of maize *pericarp color1* (*p1*) [42, 43]. P1 protein binds the *cis*-regulatory elements CCTACC (-614 to -553) and CCAACC (-83 to -78) and controls *F3′H* expression in maize [43]. However, in sorghum the promoter of *F3′H* (Sb04g024710N in Nakei-MS3B or Sb04g024710 in M36001) does not contain the consensus sequence (Additional file 3: Figure S2), suggesting that sorghum requires other MYB transcription factors for the color variation of leaf caused by infection with *B. sorghicola*.

### Diversity of *F3′H*genes

Cytochrome P450 participates in metabolic networks such as those involving anthocyanins, tannins, flavones, and isoflavonoids [44, 45]. Cytochrome P450 domain–containing genes are abundant in sorghum: 326 genes encoding cytochrome P450 enzymes are annotated in sorghum BTx623, including the longest tandem gene array (15 genes) [10]. Our combination of QTL analysis (Figure 2) and transcriptome analysis (Figures 4 and 5) was a powerful tool for identifying the key genes expressed among family members—particularly an *F3′H* gene (Sb04g024710N) expressed among P450 family members (Figure 5). Sb04g024710 (*SbF3′H2*) expression is also involved in pathogen-specific 3-deoxyanthocyanidin synthesis in sorghum mesocotyls [26]. Even though the downstream homologous gene Sb04g024750 (*SbF3′H1*) was also expressed in our study, its expression level was not as high as that of Sb04g024710N (Figure 4). Sb04g024750 (*SbF3′H1*) is expressed during light-specific anthocyanin accumulation [26]. Sb09g022480.1 had 72.3% amino acid identity to *F3′H* (Sb04g024710.1), but its expression pattern was different from that of *F3′H* (Sb04g024710.1) (Figure 5). In sorghum, these duplications have resulted in diversity of both genomic sequences and gene expression; homologous genes have thereby developed different functions on an evolutionary time scale.

In other plants, mutants in which coloration is affected are also deficient in *F3′H*. The *tt*7 mutation in Arabidopsis, which makes the seeds pale brown, is caused by a single base transition generating a stop codon [46]. The *t* mutant in soybean, which affects pigmentation in the seed coat and trichome hairs, is caused by a frameshift mutation [47, 48]. Of three spontaneous mutations in morning glory species, that on the magenta allele is a nonsense mutation generating a stop codon; pink mutants carry an insertion of the Ac/Ds superfamily transposable element; and the fuchsia allele is a single T insertion generating a stop codon [49]. All of the *F3′H* genes on mutated alleles in Arabidopsis, soybean, and morning glory encode non-functional proteins. We consider that both of our *F3′H* alleles (Sb04g024710N and Sb04g024710) were functional, as encoded proteins fully complement the *tt7* mutation in Arabidopsis [26]. Thus, the expression levels of *F3′H* genes are important for the gradual variation in color in disease-affected sorghum leaves. Coloration by flavonoids protects leaf cells from photooxidative damage, thus enhancing the efficiency of nutrient retrieval during senescence [50], and is responsible for a visual signal that attracts pollinators [37, 51]. Even though luteolinidin is toxic towards fungi and sorghum lines resistant to the fungus accumulate luteolinidin at higher levels than apigeninidin [8, 52], the biological importance of the coloration itself to the defense response against fungi remains to be elucidated.

## Conclusions

Expression of *F3′H* is responsible for the synthesis of luteolinidin; the level of expression of *F3′H* is thus a critical determinant of color variation in sorghum leaves infected with *B. sorghicola*.

## Electronic supplementary material

Additional file 1: Table S1: Primers used. (XLS 31 KB)

Additional file 2: Figure S1: Comparison of amino acid sequences of *F3′H* from Nakei-MS3B and BTx623. The *F3′H* gene of Nakei-MS3B (Sb04g04710N) is the fused gene of Sb04g04710 and Sb04g04730 shown in Figure 3A; that of BTx623 is Sb04g024710, which is annotated in Phytozome. Two amino acids (red) are substituted in Nakei-MS3B. (PDF 39 KB)

Additional file 3: Figure S2: Comparison of upstream regions of *F3′H.* Upstream regions (1000 bp) of *F3′H* in BTx623 (Sb04g04710, upper) and Nakei-MS3B (Sb04g04710N, lower) are compared. Two nucleotide substitutions (at positions -144 and -664), and one insertion/deletion (at position -661) are shown in red. Nucleotide positions are counted from the transcription start site (position 1). (PDF 44 KB)

Additional file 4: Figure S3: Nucleotide polymorphisms in *Tan1* (Sb04g031730), and the deduced amino acid sequences. *Tan1* of Shan Qui Red sorghum encodes a functional WD40 protein [34]. A 10-bp insertion in the exon causes a frame shift in M36001 and BTx623 sorghum. Nucleotide positions are based on the Shan Qui Red *tan1* gene (accession number JX122967). (PDF 49 KB)

Additional file 5: Table S2: Expression ratios and description of transcripts. Transcript ID (Transcript), gene ID (Gene), chromosome number (Chromosome), start position (Start), end position (End), strand direction (Strand), description in Phytozome (Description), pfam ID (Pfam), reads per kilobase of exon model per million mapped reads (RPKM) before (before) or after (after) cutting stress, and calculated ratio of RPKM (Fold change) in each line are listed. (XLS 15 MB)
