# Supplementary material for: Expression level of a flavonoid 3′-hydroxylase gene determines pathogen-induced color variation in sorghum
Source: BMC Res Notes. 2014 Oct 27;7:761. doi: 10.1186/1756-0500-7-761 (PMC4219097; doi:10.1186/1756-0500-7-761)
Supplement: Supplementary file 2 — Additional file 2: Figure S1: Comparison of amino acid sequences of F3′H from Nakei-MS3B and BTx623. The F3′H gene of Nakei-MS3B (Sb04g04710N) is the fused gene of Sb04g04710 and Sb04g04730 shown in Figure 3A; that of BTx623 is Sb04g024710, which is annotated in Phytozome. Two amino acids (red) are substituted in Nakei-MS3B. (PDF 39 KB) [file 13104_2014_3281_MOESM2_ESM.pdf]

Flavonoid 3'-hydroxylase (F3'H)

```
BTx623          1   MDVPLPLLLGSLAVSVVVWCLLLRRGGDGKGKGKRPMPPGPRGWPVLGNLPQLGSHPHHT
 (Sb04g024710)      ************************************************************
Nakei-MS3B      1   MDVPLPLLLGSLAVSVVVWCLLLRRGGDGKGKGKRPMPPGPRGWPVLGNLPQLGSHPHHT
 (Sb04g024710N)
               61   MCALAKKYGPLFRLRFGSAEVVVAASARVAAQFLRTHDANFSNRPPNSGAEHVAYNYQDM
                    ************************************************************
               61   MCALAKKYGPLFRLRFGSAEVVVAASARVAAQFLRTHDANFSNRPPNSGAEHVAYNYQDM

              121   AFAPYGSRWRALRKLCALHLFSAKALDDLRSIREGEVALLVRELSRHQHQHAGVPLGQVA
                    ************************************************************
              121   AFAPYGSRWRALRKLCALHLFSAKALDDLRSIREGEVALLVRELSRHQHQHAGVPLGQVA

              181   NVCATNTLARATVGRRVFAVDGGEEAREFKDMVVELMQLAGVFNVGDFVPALARLDLQGV
                    ************************************************************
              181   NVCATNTLARATVGRRVFAVDGGEEAREFKDMVVELMQLAGVFNVGDFVPALARLDLQGV

              241   VGKMKRLHRRYDDMMNGIIRERKAAEEGKDLLSVLLARTREQQSIADGEDSRITETEIKA
                    ************************************************************
              241   VGKMKRLHRRYDDMMNGIIRERKAAEEGKDLLSVLLARTREQQSIADGEDSRITETEIKA

              301   LLLNLFTAGTDTTSSTVEWALAELIRHPDVLKKAQEELDAVVGRNRLVSELDLPRLTYLT
                    ************************************************************
              301   LLLNLFTAGTDTTSSTVEWALAELIRHPDVLKKAQEELDAVVGRNRLVSELDLPRLTYLT

              361   AVIKETFRMHPSTPLSLPRIAAEECEVDGFRIPAGTTLLVNVWAIARDPEAWPEPLQFRP
                    ************************************************************
              361   AVIKETFRMHPSTPLSLPRIAAEECEVDGFRIPAGTTLLVNVWAIARDPEAWPEPLQFRP

              421   DRFLPGGSHAGVDVKGSDFELIPFGAGRRICAGLSWGLRMVTLMTATLVHALDWDLADGM
                    ************************************************************
              421   DRFLPGGSHAGVDVKGSDFELIPFGAGRRICAGLSWGLRMVTLMTATLVHALDWDLADGM

              481   TADKLDMEEAYGLTLQRAVPLKVRPAPRLLPSAYAAE    (518aa)
                    ********************* *** ***********
              481   TADKLDMEEAYGLTLQRAVPLMVRPTPRLLPSAYAAE    (518aa)
```

Figure S1
