# Supplementary material for: Expression level of a flavonoid 3′-hydroxylase gene determines pathogen-induced color variation in sorghum
Source: BMC Res Notes. 2014 Oct 27;7:761. doi: 10.1186/1756-0500-7-761 (PMC4219097; doi:10.1186/1756-0500-7-761)
Supplement: Supplementary file 3 — Additional file 3: Figure S2: Comparison of upstream regions of F3′H. Upstream regions (1000 bp) of F3′H in BTx623 (Sb04g04710, upper) and Nakei-MS3B (Sb04g04710N, lower) are compared. Two nucleotide substitutions (at positions -144 and -664), and one insertion/deletion (at position -661) are shown in red. Nucleotide positions are counted from the transcription start site (position 1). (PDF 44 KB) [file 13104_2014_3281_MOESM3_ESM.pdf]

```
BTx623  -1002 TGAGAACTAAAACTAATAGCACAATTTGTCTGTAAATTACGAGACGAATCTTTTAAACCT
              ************************************************************
Nakei   -1002 TGAGAACTAAAACTAATAGCACAATTTGTCTGTAAATTACGAGACGAATCTTTTAAACCT

         -942 AGTTACTTTATGATTAGATAATGTTTGTCAAGTAAAAACGAAAATATTATAGTGTCAAAA
              ************************************************************
         -942 AGTTACTTTATGATTAGATAATGTTTGTCAAGTAAAAACGAAAATATTATAGTGTCAAAA

         -882 TCCAAAAACTTTTTGAATCTAAATAAGGCCCGCGTTGAAAATGACATAGAATTGATTGTA
              ************************************************************
         -882 TCCAAAAACTTTTTGAATCTAAATAAGGCCCGCGTTGAAAATGACATAGAATTGATTGTA

         -822 CTGTTGCTGGGCACTGCTGCTCACTGCACGACTCCTCGTTCTGTTAATGATTTTGTGAAA
              ************************************************************
         -822 CTGTTGCTGGGCACTGCTGCTCACTGCACGACTCCTCGTTCTGTTAATGATTTTGTGAAA

         -762 ATTGATTACACCGCTATTGAGCACTGCTGATTGCTGAGCACTGCCGCTCTCAAATGATTT
              ************************************************************
         -762 ATTGATTACACCGCTATTGAGCACTGCTGATTGCTGAGCACTGCCGCTCTCAAATGATTT
                                                    (-664) (-661)
         -702 TGTGGAAACAGTACATTTGAAATAAAATTATTTTGTGGGTCTTTTTTTTTTAAATTTTGCC
              ************************************** ** ******************
         -702 TGTGGAAACAGTACATTTGAAATAAAATTATTTTGTGGCTC-TTTTTTTTAAATTTTGCC

         -642 AATTCAAAATTGCCAAATTCTTAGAAATAAGATGATCTTTTACTTGGCATATATTTTTAG
              ************************************************************
         -642 AATTCAAAATTGCCAAATTCTTAGAAATAAGATGATCTTTTACTTGGCATATATTTTTAG

         -582 GAGTTGGCAAATCATATCACAGAGTTTCTTCGATCTTGTGTCGAAGCCGGCTATAAGCTT
              ************************************************************
         -582 GAGTTGGCAAATCATATCACAGAGTTTCTTCGATCTTGTGTCGAAGCCGGCTATAAGCTT

         -522 GCAGCCTTCTTCTTATTTTTCTCTTTTTTTTTCTTTTTTGTTCTCTTTTTCCTCGTTTTT
              ************************************************************
         -522 GCAGCCTTCTTCTTATTTTTCTCTTTTTTTTTCTTTTTTGTTCTCTTTTTCCTCGTTTTT

         -462 GTCCTCCATTTCAGTATTTAGCCAATTTACAAACTGTTATTATACTTGCTCTTTTATCTC
              ************************************************************
         -462 GTCCTCCATTTCAGTATTTAGCCAATTTACAAACTGTTATTATACTTGCTCTTTTATCTC

         -402 CTACTCCCTCCGTCCCTGAAAGCTGCAATTTCTAGAGTTTTTCTAAGTCAATTTTTTACA
              ************************************************************
         -402 CTACTCCCTCCGTCCCTGAAAGCTGCAATTTCTAGAGTTTTTCTAAGTCAATTTTTTACA

         -342 ACTTTGACCAAATTTATAGAAAAAAAATGCTAAGATTTATAGTACCAAATTAATACCATTA
              ************************************************************
         -342 ACTTTGACCAAATTTATAGAAAAAAAATGCTAAGATTTATAGTACCAAATTAATACCATTA

         -282 AATTTATTATAGAATATATTTTTGAAAGATACTTATTTTATGTTATAGATATTGATACTC
              ************************************************************
         -282 AATTTATTATAGAATATATTTTTGAAAGATACTTATTTTATGTTATAGATATTGATACTC

         -222 TTTTCTATAAAGTTGGTCAAAGTTAGAAAAGTTTGACTCACATGGATTCTAGGAGTTGAA
              ************************************************************
         -222 TTTTCTATAAAGTTGGTCAAAGTTAGAAAAGTTTGACTCACATGGATTCTAGGAGTTGAA
                              (-144)
         -162 GCTTTTGGGGACGGACCACTAAAGTCCTCCGAGCTCCCCAATTGCGGCGTGGGTAGGTAG
              ****************** *****************************************
         -162 GCTTTTGGGGACGGACCAGTAAAGTCCTCCGAGCTCCCCAATTGCGGCGTGGGTAGGTAG

         -102 GGACGGCGCCTCAGGGAGATTGTTTGCTGAAGCCTGAAGCACGCCAAGTGAAGTGGCATG
              ************************************************************
         -102 GGACGGCGCCTCAGGGAGATTGTTTGCTGAAGCCTGAAGCACGCCAAGTGAAGTGGCATG
                                             --> Transcription
          -42 TGTATATATAGATGAACCACTCCCACGCTAACCGCACGCTCAACGCTCAGTCCAACGCCA
              ************************************************************
          -42 TGTATATATAGATGAACCACTCCCACGCTAACCGCACGCTCAACGCTCAGTCCAACGCCA
                                           (1)
```

Figure S2
