# Supplementary material for: Expression level of a flavonoid 3′-hydroxylase gene determines pathogen-induced color variation in sorghum
Source: BMC Res Notes. 2014 Oct 27;7:761. doi: 10.1186/1756-0500-7-761 (PMC4219097; doi:10.1186/1756-0500-7-761)
Supplement: Supplementary file 4 — Additional file 4: Figure S3: Nucleotide polymorphisms in Tan1 (Sb04g031730), and the deduced amino acid sequences. Tan1 of Shan Qui Red sorghum encodes a functional WD40 protein [34]. A 10-bp insertion in the exon causes a frame shift in M36001 and BTx623 sorghum. Nucleotide positions are based on the Shan Qui Red tan1 gene (accession number JX122967). (PDF 49 KB) [file 13104_2014_3281_MOESM4_ESM.pdf]

Tan1 Sb04g031730

```
                               901       911       921       931
             Nakei-MS3B        TGGGAACTGCCCGAGACGGCGGCGGCTGTGCCCGCCGAG
Tan1         ShanQuiRed        TGGGAACTGCCCGAGACGGCGGCGGCTGTGCCCGCCGAG

             (amino acid)       W  E  L  P  E  T  A  A  A  V  P  A  E

             M36001            TGGGAACTGCCCGAGACGGCGGCGGGCAGCGGCGGCTGT
tan1b        BTx623            TGGGAACTGCCCGAGACGGCGGCGGGCAGCGGCGGCTGT
                                                     10bp-insertion
             (amino acid)       W  E  L  P  E  T  A  A  G  S  G  G  C
                                                       frame shift
```

In the M36001 and BTx623 sequences, the 10-bp insertion **CGGGCAGCGG** is printed in bold and underlined. In the tan1b amino-acid line, A G S G G C is underlined and labelled "frame shift".

Figure S3
